# ^18^F-DCFPyL (PSMA) PET in the Management of Men with Biochemical Failure after Primary Therapy: Initial Clinical Experience of an Academic Cancer Center

**DOI:** 10.3390/curroncol28050282

**Published:** 2021-08-25

**Authors:** Ur Metser, Claudia Ortega, Douglas Hussey, Rosanna Chan, Alejandro Berlin, Antonio Finelli, Patrick Veit-Haibach

**Affiliations:** 1 Joint Department of Medical Imaging, University Health Network, Mount Sinai Hospital & Women’s College Hospital, Toronto, ON M5G2M9, Canada; claudia.ortega@uhn.ca (C.O.); douglas.hussey@uhn.ca (D.H.); rosanna.chan@uhn.ca (R.C.); patrick.veit-haibach@uhn.ca (P.V.-H.); 2 Department of Medical Imaging, University of Toronto, Toronto, ON M5T 1W7, Canada; 3 Princess Margaret Cancer Center, Department of Radiation Oncology, University Health Network, Toronto, ON M5G 2M9, Canada; alejandro.berlin@rmp.uhn.ca; 4 Department of Radiation Oncology, University of Toronto, Toronto, ON M5T 1P5, Canada; 5 Princess Margaret Cancer Centre, Division of Urology, Department of Surgery, University Health Network, Toronto, ON M5G 2M9, Canada; Antonio.Finelli@uhn.ca

**Keywords:** ^18^F-DCFPyL, PET/CT, PET/MRI, biochemical recurrence, prostate cancer

## Abstract

Purpose: To describe the initial experience of an academic center using ^18^F-DCFPyL PET in managing men with recurrent prostate cancer. Materials & Methods: This prospective, single-arm IRB-approved study included men with biochemical failure after primary therapy for prostate cancer and negative/equivocal CT and bone scintigraphy who were candidates for salvage therapy, as determined by a multidisciplinary panel of experts. ^18^F-DCFPyL PET was assessed for the presence and extent of recurrence: local, oligometastatic (≤4), or extensive. Post-PET management and clinical outcome, including PSA response, was documented. For patients who received PET-directed ablative therapies, response was categorized as “complete” if PSA became undetectable or “favorable” if PSA decreased ≥50%. Results: Forty-seven men with biochemical failure after radical prostatectomy (*n* = 29), primary radiotherapy (*n* = 15) or focal tumor ablation (*n* = 3) were included. PET was positive in (43/47) 91.5%, including local recurrence in (9/47) 19.2%; oligometastatic disease in (16/47) 34%; and extensive metastatic disease in (18/47) 38.3%. PET-directed focal ablative therapies without systemic therapy were given to (13/29) 44.8% of patients without extensive metastases on PET with a mean PSA response of 69% (median, 74.5%; range: 35–100). Favorable biochemical response was observed in (10/13) 76.9% of patients with limited recurrence on PET, and in 23.1% (3/13), there was complete response. Conclusion: ^18^F-DCFPyL PET was positive in >90% of patients with biochemical failure. For those with limited recurrence, PSMA PET-directed local ablative therapies resulted in favorable outcome in more than 3 in 4 patients, and in nearly a quarter of them, there was complete biochemical response.

## 1. Introduction

Biochemical failure (BCF) after primary therapy for prostate cancer can occur within 10 years in 20–40% of men who undergo radical prostatectomy and 30–50% of men who undergo definitive radiotherapy [1,2,3]. Although BCF indicates recurrent disease, this may have little impact on the patient’s overall survival or quality of life. The clinical challenge in managing BCF is to prevent or delay the onset of metastatic disease while considering the morbidity associated with the various therapies and their impact on quality of life. At time of BCF, localization and extent of disease is crucial to customizing a therapeutic approach. Although there is currently no consensus regarding the optimal management of patients with BCF, localized tumor recurrence offers the opportunity to treat patients with local salvage and delay salvage systemic therapies. These therapeutic strategies may include salvage radiotherapy in patients with BCF after radical prostatectomy or with stereotactic ablative radiotherapy (SABR) or surgery for limited metastases [4]. This strategy may delay the need for salvage androgen deprivation therapy and the significant complications associated with it [4,5]. 

PSMA PET has been evaluated in recent years for the restaging of patients with prostate cancer at time of BCF with encouraging results. The purpose of this manuscript is to describe the initial clinical experience of an academic center using ^18^F-DCFPyL (PSMA) PET in managing men with recurrent prostate cancer and its impact on patient outcome. 

## 2. Patients & Methods

This is a prospective, single-arm research ethics board-approved registry study (NCT03535831). Informed consent was obtained from all participants. The current analysis is of men with histologically proven prostate cancer after primary therapy, with biochemical failure and negative or equivocal CT and bone scintigraphy within 4 months of enrollment. All patients were not receiving androgen deprivation therapy at time of enrollment. After initial screening by a study coordinator, all relevant clinical and imaging data of potential study subjects were reviewed prospectively and before inclusion by a multidisciplinary panel including a nuclear medicine physician/radiologist, surgeon, radiation oncologist and medical oncologist. The panel assessed whether the patient would potentially be eligible for local or potentially focal salvage therapies if local or limited metastatic disease was identified and determined the patient’s management plan prior to PET. 

### 2.1. Study Procedures

The choice of imaging platform, PET/CT or PET/MR was made according to the need for concurrent prostate or prostate fossa MR. Specifically patients who did not have pelvic MR within 6 months of registration and who had a clinical indication to evaluate the prostate or prostate fossa with no contraindications for MR imaging received PET/MR; all others received PET/CT. 

PET/CT or PET/MR was performed 110.2 (±13.9) minutes following injection of 327 (±16.7) MBq of ^18^F-DCFPyL. Patients were positioned supine on the imaging couch with arms outside of the region of interest. Images were obtained from the top of the skull to the upper thighs. PET/CT was performed on a Biograph mCT 40 scanner (Siemens Healthcare, Erlangen, Germany). CT was used for attenuation correction as per standard PET/CT departmental protocols. Overall, 5–9 bed positions were obtained as per patient height (2–5 min/bed position). PET/MR was performed on an integrated PET/MRI system (Biograph mMR, Siemens Healthcare). For PET/MR, a whole-body protocol previously described was used, including coronal Dixon sequences (5–6 bed positions; 2–5 min/bed position) for attenuation correction and whole-body gadolinium-enhanced T1-weighted volumetric interpolated breath-hold examination (VIBE) [6]. PET/CT or PET/MR were interpreted prospectively by one of 4 radiologists/nuclear medicine physicians with 10–22 years of experience reporting PET using the previously described Prostate Cancer Molecular Imaging Standardized Evaluation (PROMISE) classification with PSMA uptake stratified by a comparison to physiological uptake in reference tissues using a 3-point scale (PSMA score) [7]. PSMA PET results were made available to the treating oncologist. 

### 2.2. Data Abstraction

Demographic data including age, tumor grade at diagnosis (International Society of Urological Pathology [ISUP] Grade Group), initial management of prostate cancer, time to biochemical recurrence and serum PSA prior to PET were recorded. Results of previously acquired CT and bone scintigraphy were tabulated. PET data was tabulated according to the following criteria: 1. Overall positivity (any positive lesion identified); 2. Location and extent of recurrence: local recurrence in the prostate or prostate fossa, oligometastatic disease defined as ≤4 sites of disease [8] or extensive disease if 5 or more sites of recurrence. Patient management was determined by the treating oncologist with all available clinical data and in discussion with the patient. Patient actual management received after PET and clinical outcome including serum PSA response were recorded. For patients who received PET-directed ablative therapies such as surgery or stereotactic radiotherapy, response to therapy was categorized as complete biochemical response if serum PSA became undetectable, favourable biochemical response if serum PSA decreased by ≥50% from pre-PET values or incomplete response if PSA decreased by <50%. 

## 3. Results

**Figure 1:**
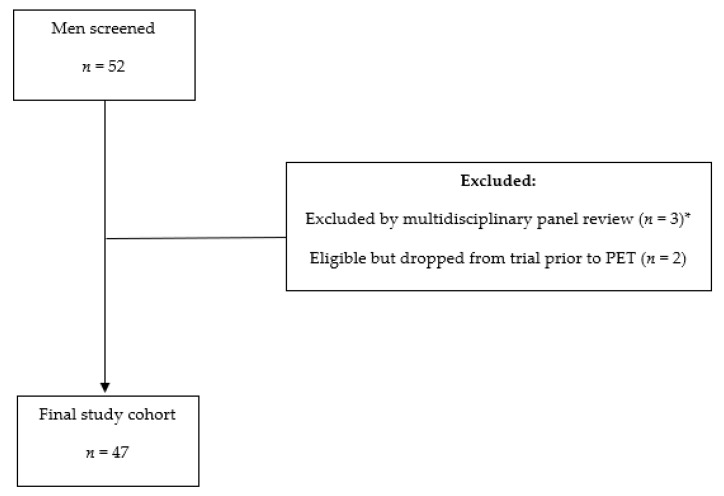
Flowchart: Subject enrollment process. * = Multidisciplinary panel consensus was that patient would not be eligible for local or focal ablative therapy if found to have limited metastatic disease.

There were 47 men with biochemical failure after radical prostatectomy (*n* = 29), primary radiotherapy (*n* = 15) or focal tumor ablation (*n* = 3) included in this analysis. Figure 1 depicts the enrollment process and the excluded men. Demographic data is summarized in Table 1. Baseline conventional imaging, including bone scintigraphy and CT, performed 9 ± 5.6 weeks prior to PET was negative in 39 patients and equivocal in 8 patients. Equivocal lesions included bone lesion on CT (*n* = 3) or bone scintigraphy (*n* = 2) or equivocal lymph node on CT (*n* = 3). 

### 3.1. PET Imaging Results

**Figure 2:**
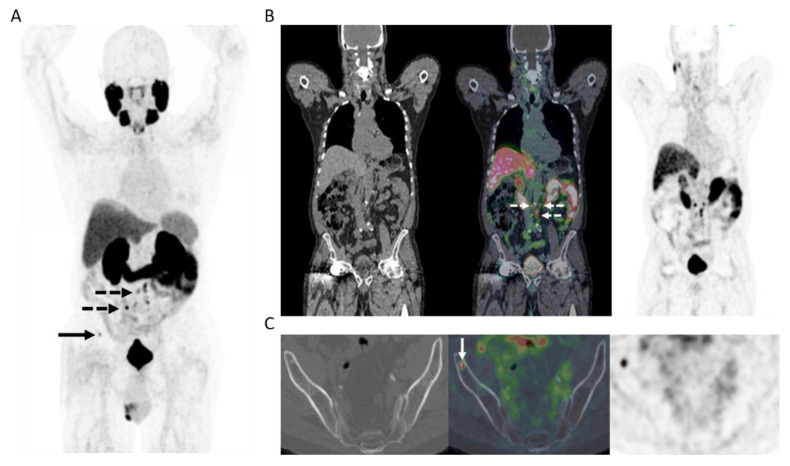
65-year-old man 12 years post low-dose rate brachytherapy for cT2 ISUP Grade Group 2 adenocarcinoma of prostate. Biochemical failure (serum PSA = 19.9 ng/mL) with negative CT and bone scintigraphy. (**A**) MIP PET image showing extensive, multifocal abnormal radiotracer uptake in the pelvis and retroperitoneum (solid arrows), in the left supraclavicular fossa (dotted arrow), as well as a few sites in the skeleton (arrowheads). (**B**) Axial PET/MR image at pelvic inlet (PET–left; gadolinium-enhanced T1-weighted image–middle; fused PET/MR–right) shows an intensely PSMA avid right common iliac node (solid arrow) and a PSMA avid deposit at L5 vertebral body (arrowhead). (**C**) Axial PET/MR image at pelvic inlet (PET–left; gadolinium-enhanced T1-weighted image–middle; fused PET/MR–right) shows a further PSMA avid bone deposit in the scapula. After PET, the patient commenced androgen deprivation therapy.

^18^F-DCFPyL PET/MR was performed in 12 patients who had a clinical need for concurrent MR of the prostate, as defined above, predominantly patients post prior radiotherapy or focal tumor ablation. In all others (*n* = 35), ^18^F-DCFPyL PET/CT was obtained. Overall, PET/MR was positive in 11/12 and PET/CT was positive in 32/35 men, with overall 43 of 47 study patients (91.5%) having at least one site of residual or recurrent disease on PET. Sites of recurrence included local tumor recurrence in the prostate or prostate bed in (20/47) 42.6%, regional nodal metastases in (29/47) 61.7%, extra-regional nodal metastases in (19/47) 40.4% or skeletal metastases in (3/47) 6.4%. Local recurrence, lymph node metastases and bone metastases were histologically confirmed in 2, 3 and 2 men, respectively. Recurrence was limited to the prostate or prostate bed only in (9/47) 19.2%, oligometastatic disease in (16/47) 34% or extensive metastatic disease in (18/47) 38.3% (Figure 2). Post PET management data is summarized in Table 2.

### 3.2. Post-PET Management & Clinical Outcome

**Figure 3:**
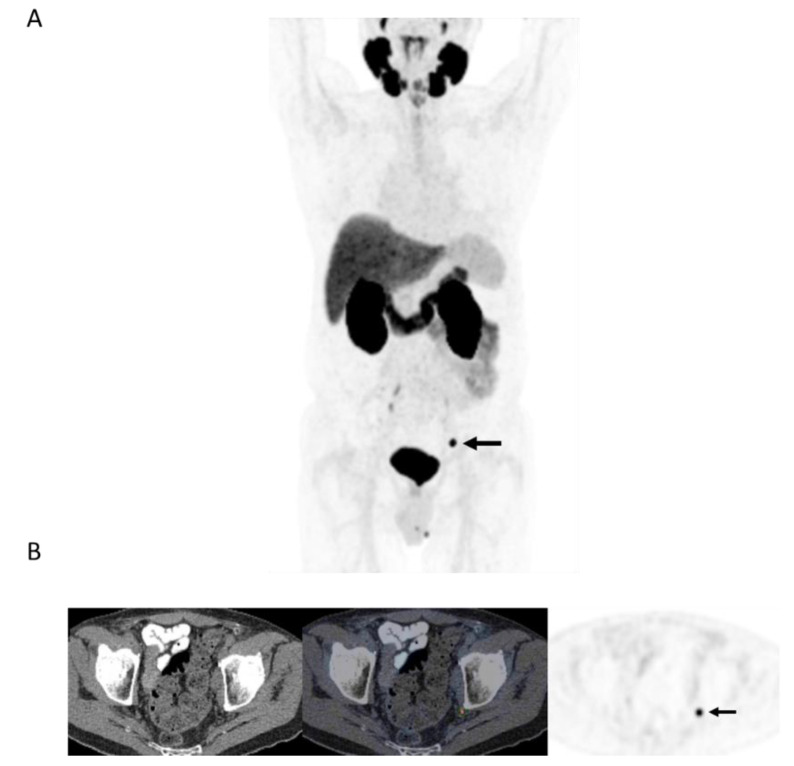
78-year-old man 4 years after radical prostatectomy for pT3a N0 adenocarcinoma of prostate ISUP Grade Group 3. Now biochemical recurrence (serum PSA = 0.80 ng/mL); PET performed prior to salvage prostate bed radiotherapy. (**A**) MIP PET image showing solitary focus of abnormal tracer uptake in the pelvis on the left. (**B**) Axial PET/CT image (CT–left; fused PET/CT–middle; PET–right) shows an intensely ^18^F-DCFPyL-avid 0.3 cm left pelvic sidewall lymph node (SUVmax = 25.6; PSMA score, 3), in keeping with a metastatic node. After PET, the patient was treated with PET-directed stereotactic radiotherapy with favorable metabolic response (>76% decrease in serum PSA).

Overall, (13/29) 44.8% of patients without extensive metastases on PET were treated with PET-directed focal ablative therapies without systemic therapy (Figure 3). The mean PSA response to focal ablative therapies was 69% (median, 74.5%; range: 35–100). Favorable biochemical response (>50% drop in pre-PET PSA) was observed in 10/13 (76.9%) patients with limited recurrence on PET, and in 3/13 (23.1%), there was complete biochemical response.

## 4. Discussion

Our initial experience with PSMA PET in the workup of patients with biochemical failure after primary therapy for prostate cancer with no definitive metastatic disease on conventional workup has shown PSMA PET positivity in more than 90% of patients, with local recurrence or oligometastatic disease in over half of them. Tumor detection rate in our cohort (with median serum PSA of 3 ng/mL) are in line with those reported in the previous prospective CONDOR trial in which disease detection was 73.3% when serum PSA was <0.5 ng/mL and rose to 96.4% when serum PSA was >5 ng/mL [9]. More than 40% of the patients with limited recurrence were treated with PET-directed ablative therapies to sites of disease without systemic therapy, and the biochemical response was favorable in more than 3 in 4 of them (Figure 3). Overall, in more than 20% of patients in this study (10/47) with biochemical failure and no conventional workup evidence of recurrent disease, PET identified limited recurrence amenable to focal ablative therapies with a favorable biochemical response (>50% drop in serum PSA). In nearly a quarter of patients treated with limited ablative therapies, there was complete biochemical response to therapy. These findings are in line with a prior prospective trial that included 72 patients in whom 38 were treated for oligorecurrent prostate cancer amenable to metastasis-directed therapy. Overall response rate in that study was 60%, including 22% with complete biochemical response at a median follow-up of 15.9 months [10]. 

The diagnostic performance of PSMA PET in this study and its impact on patient management and outcome are in line with results from prior clinical trials. A recent meta-analysis assessing the performance of PSMA PET in the biochemical recurrence setting, which included 37 manuscripts and a total of 4790 patients, has shown that the positivity rate of PSMA PET increases with serum PSA and was 95% when PSA was 2 ng/mL or greater [9], similar to that observed in our cohort. The main clinical utility of PSMA PET in men with biochemical recurrence is to stratify patients who have local recurrence or limited metastatic disease that may be amenable to focal ablative therapies from those who have more extensive metastatic disease and would benefit from systemic therapy. Prior studies have shown a change in intended or actual management in between a third to two-thirds of patients [11,12,13,14]. Multiple further studies assessed the impact of PET on patient outcome [15,16,17,18,19,20,21,22]. A prior prospective study documented a PSA response of > 50% in nearly 3 of 4 patients (63 of 86; 73.3%), much like the results from our registry, and the 3-year biochemical free survival was 55% [15].

While this study was prospective, it was designed as a data collection registry. The heterogeneous cohort of patients in the registry reflected the patient population with biochemical failure encountered in a large academic cancer center. Patient selection was through consensus at multidisciplinary tumor board discussion, considering clinical parameters, including patient history, conventional imaging workup and comorbidities, likely reflecting the cohort of patients that would potentially benefit from more accurate staging. PSMA PET interpretation was performed locally by one of several experienced readers. Although histological confirmation was not mandated, and often not possible, PSMA PET has been shown to have very high specificity [10]. Patient management was not mandated and was provided by the treating oncologists after discussion with the patient. Therefore, we believe the results of the current study are a snapshot of the potential real-world impact of PSMA PET if incorporated into routine clinical practice and confirm the observations from prior trials on the impact of PET on the outcomes of men with biochemical failure after primary therapy for prostate cancer. Data on long-term oncological outcome and overall survival are pending. 

In conclusion, 18F-DCFPyL PET was positive in >90% % of patients with biochemical recurrence and allowed the stratification of patients to limited versus disseminated disease relapse. For patients with limited recurrence, PSMA PET-directed local ablative therapies resulted in a favourable outcome in more than 3 in 4 patients, and in nearly a quarter of them, complete biochemical response was documented. The results from this prospective PET registry were in line with those of prior prospective trials, further confirming the impact of PSMA PET on patient outcome.

## Figures and Tables

**Table 1:** Demographic data.

Title	Range	Median
Age (years)	50–83	68
ISUP Grade Group (at diagnosis)	1–5	3
Time to biochemical failure (months)	0–144	36
Serum PSA ng/mL	0.5–43.4	3.0

PSA = prostate-specific antigen.

**Table 2:** Patient management according to patient cohorts by ^18^F-DCFPyL PET results.

COHORT (*n*)	Mode of Therapy	N= (%)
**Negative PET or Local recurrence only** **(*n* = 13)**	Systemic only	2
		(15.4%)
	PET-directed ablative therapy *	6
		(46.1%)
	Observation	5
		(38.5%)
**Oligometastatic disease** **(*n* = 16)**	Systemic only	5
		(31.3%)
	PET-directed ablative therapy	8
		(50%)
	Observation	3
		(18.7%)
**Extensive metastases** **(*n* = 18)**	Systemic only	16
		(88.9%)
	PET-directed ablative therapy	0
		(0%)
	Observation	2
		(11.1%)

* = One patient received PET-directed ablative therapy and androgen deprivation therapy.

## Data Availability

Study data is available in local research databases.
